# A systematic, large-scale comparison of transcription factor binding site models

**DOI:** 10.1186/s12864-016-2729-8

**Published:** 2016-05-21

**Authors:** Daniela Hombach, Jana Marie Schwarz, Peter N. Robinson, Markus Schuelke, Dominik Seelow

**Affiliations:** Department of Neuropaediatrics, Charité—Universitätsmedizin Berlin, Berlin, Germany; NeuroCure Clinical Research Center, Charité – Universitätsmedizin Berlin, Berlin, Germany; Institute for Medical Genetics and Human Genetics, Charité—Universitätsmedizin Berlin, Berlin, Germany; Berliner Institut für Gesundheitsforschung / Berlin Institute of Health, Berlin, Germany

**Keywords:** Transcription factor binding sites, TFBS prediction, PSSM, Genetic variation

## Abstract

**Background:**

The modelling of gene regulation is a major challenge in biomedical research. This process is dominated by transcription factors (TFs) and mutations in their binding sites (TFBSs) may cause the misregulation of genes, eventually leading to disease. The consequences of DNA variants on TF binding are modelled *in silico* using binding matrices, but it remains unclear whether these are capable of accurately representing in vivo binding.

In this study, we present a systematic comparison of binding models for 82 human TFs from three freely available sources: JASPAR matrices, HT-SELEX-generated models and matrices derived from protein binding microarrays (PBMs). We determined their ability to detect experimentally verified “real” in vivo TFBSs derived from ENCODE ChIP-seq data. As negative controls we chose random downstream exonic sequences, which are unlikely to harbour TFBS. All models were assessed by receiver operating characteristics (ROC) analysis.

**Results:**

While the area-under-curve was low for most of the tested models with only 47 % reaching a score of 0.7 or higher, we noticed strong differences between the various position-specific scoring matrices with JASPAR and HT-SELEX models showing higher success rates than PBM-derived models. In addition, we found that while TFBS sequences showed a higher degree of conservation than randomly chosen sequences, there was a high variability between individual TFBSs.

**Conclusions:**

Our results show that only few of the matrix-based models used to predict potential TFBS are able to reliably detect experimentally confirmed TFBS.

We compiled our findings in a freely accessible web application called ePOSSUM (http:/mutationtaster.charite.de/ePOSSUM/) which uses a Bayes classifier to assess the impact of genetic alterations on TF binding in user-defined sequences. Additionally, ePOSSUM provides information on the reliability of the prediction using our test set of experimentally confirmed binding sites.

**Electronic supplementary material:**

The online version of this article (doi:10.1186/s12864-016-2729-8) contains supplementary material, which is available to authorized users.

## Background

Transcription factors (TFs) are major players in the regulation of gene activity. They bind to DNA target sequences known as transcription factor binding sites (TFBSs) in order to up- or down-regulate transcriptional activity. As DNA binding is pivotal to correct TF function, mutations in TFBSs can lead to genetic disease [1, 2].

However, despite the rapid growth in the number of mutations known to cause Mendelian disorders, the number of confirmed mutations in regulatory sequences is still low, presumably due to difficulties in experimentally verifying their functional relevance as the cause of a given disease.

With each deep sequencing run a large number of DNA variants are found, many of which may be considered as potentially causing Mendelian disorders, even after bioinformatic filtering. Hence the main challenge is currently not the generation, but the interpretation of the data. While considerable progress has been made at the *in silico* analysis of alterations in the protein coding sequences (e.g. MutationTaster [3], SIFT [4], PolyPhen-2 [5]), these prediction tools address the role of variants within potential regulatory sequences only indirectly, e.g. via their evolutionary conservation, or the simple fact that a variant occurred somewhere within a confirmed regulatory element. We thus set out to examine and compare the relevance of methods for computational TFBS recognition as a means to assess the disease-causing potential of genetic variants.

Currently, the state-of-the art method for functionally representing TFBSs is via transcription factor binding models that are described by matrices, such as position-specific scoring matrices (PSSMs), also called position weight matrices (PWMs). These PSSMs are normalised representations of the position-specific log-likelihoods of a nucleotide’s probability to occur at each location in an observed sequence [6]. PSSMs can be used to computationally detect putative TFBSs in regulatory regions by establishing a match score, which determines how well a given sequence matches with a TF binding model.

A manually curated census in the year 2009 counted 1391 TFs in humans [7]. This number has grown since then—estimates for the total number of human TFs currently range between 2000 and 3000 [7, 8]. However, only a fraction of these factors have been studied intensely enough to generate reliable binding models; the ENCODE (Encyclopaedia of DNA Elements) project, for example, offers one of the largest collections of experimentally verified binding instances, but only covers 161 different TFs [9].

Moreover, various other repositories of TF binding models exist that have been obtained by different methods, but still fall short to represent the predicted TF landscape in humans. The largest of these repositories, TRANSFAC [10], is not freely available and has therefore not been included into this survey. The human Protein-DNA Interactome (hPDI) [11] contains matrices for 493 human TFs generated through Protein-Binding Microarrays (PBMs), whereas a recent publication [12] determined over 200 matrices for 151 human TFs via an HT-SELEX experiment. HT-SELEX enables the identification of DNA target strands through a large library of artificially generated oligonucleotides, which are presented to a target protein. Bound sequences are amplified by PCR, followed by several rounds of selection processes to determine the best-bound sequences. It is not surprising that binding models derived from different experimental methods can show highly different success rates in modelling TF-DNA binding. However, for a long time it was impossible to compare the quality of binding models that had been obtained by various methods due to the lack of sufficient experimental data.

Therefore, only recently a direct comparison between TF models derived from PBM and HT-SELEX-experiments has been published [13]. This study found models generated through both experimental strategies to be quite similar for in vitro binding, whereas for in vivo prediction HT-SELEX-derived models achieved better results.

Here we present a larger study in which we set out to compare a total of 179 PSSMs linked to 82 different TFs that derived from the following three sources: HT-SELEX, PBM, and the manually curated binding models provided by JASPAR [14]. Almost all of the 58 JASPAR matrices (representing 56 TFs) used in our study resulted from ChIP-seq experiments, only two, aimed at NFIC and RELA, were generated by HT-SELEX experiments. We tested all models for their capability to recognise ChIP-seq confirmed functional (‘*in vivo*’) TFBSs from the ENCODE project [15]. Due to the presence of several TFs in more than one binding model source, we were able to directly compare matrices linked to 26 different TFs from JASPAR and HT-SELEX, thereby enabling a quality assessment of binding models obtained through different experimental methods. For this study, we created two test sets, one comprising all suitable TFBSs from ENCODE and another one which includes only those TFBSs with a high binding score (‘high-confidence set’). We used the same number of length-matched exonic sequences as a TFBS-depleted control set.

This study is to our knowledge the largest comparison of TF binding models, testing 179 binding models (PSSMs) on over 2 x 2,000,000 target sequences in total. Hence, our study provides insight into the current quality of computational TFBS predictions and the findings generated in this study may help to evaluate the feasibility of whole-genome TFBS prediction studies.

## Results and discussion

### Binding predictions

We tested the PSSMs mentioned above and used ChIP-seq derived sequences binding to TFs as positive controls. Results were assessed with ROC analysis (see Methods for details).

The various binding models from the multiple sources yielded different results in many cases: While only 3 of the 19 PBM-derived models from hPDI (16 %) reached an AUC score of ≥0.7, this was the case for 46 % of the HT-SELEX and 60 % of the JASPAR-models. The average AUC values generated by the different binding model sources were highly divergent, ranging from 0.53 (hPDI) to 0.72 (JASPAR).

**Fig. 1:**
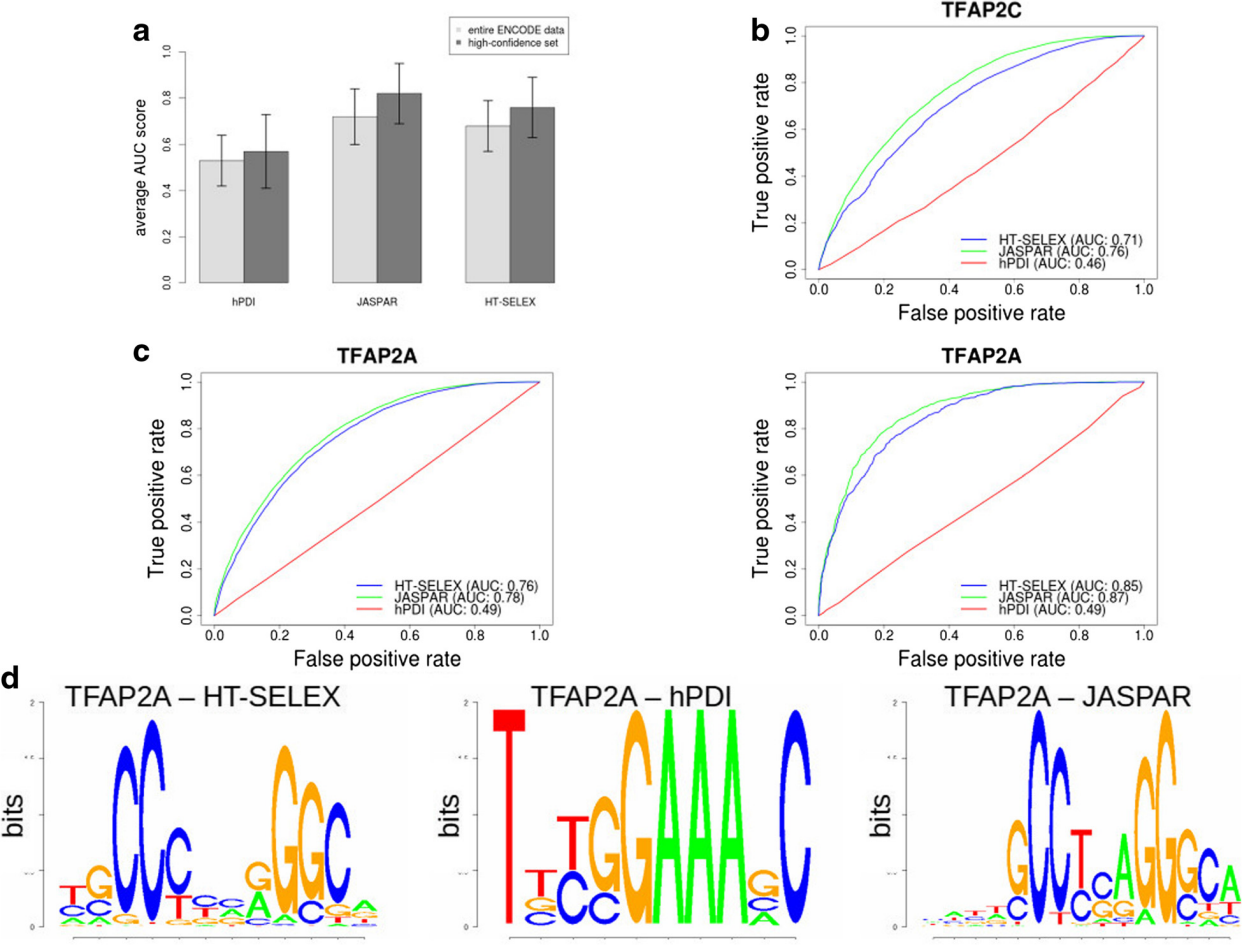
Average AUC scores and representative ROC plots for different binding model sources. **a** Average AUC scores generated for the different binding model sources. **b** ROC plot for TFAP2C. **c** ROC plots for TFAP2A for the entire ENCODE test set (*left*) and the high confidence set (*right*). **d** Underlying TF binding models for TFAP2A

When tested on the ‘high-confidence’ dataset, 4 out of 15 models (26.6 %) from hPDI yielded AUC scores of ≥0.7, which resulted in an average AUC score of 0.57. The HT-SELEX-models reached AUC values ≥0.7 in 70 % of the cases (average AUC of all PSSMs: 0.76), whereas the JASPAR-matrices generated an average AUC score of 0.83. An overview of these findings is depicted in Fig. 1.

**Fig. 2:**
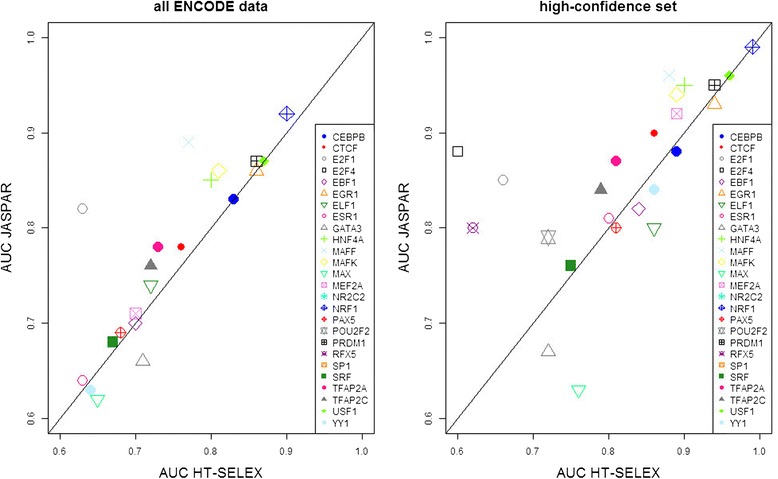
Direct comparison of binding models generated by different methods. Depicted are AUC scores for TFs stored in both JASPAR (manually collected curated models) and HT-SELEX. AUC scores were generated using ROCR. If multiple binding models were available for one TF, we depict the average AUC value

We were able to directly compare the prediction quality of models for 28 different TFs that were represented in both the JASPAR and the HT-SELEX data (26 for the high confidence test set). We found that in general, the manually curated matrices from JASPAR enabled a slightly better distinction between positive and negative sequences (average AUC 0.74 versus 0.70). In the ‘high-confidence’ data set, these values increased for both data model sources: The HT-SELEX models generated an average AUC of 0.80, whereas for JASPAR this value was 0.84 (Figs. 1a and 2).

These findings can help to estimate the utility of TFBS models from different sources, enabling researchers to better circumvent the high false-positive rate, a problem which is well known for *in silico* TF binding analyses [16], and thus to make the most of the available data. In general, we found manually curated models provided by JASPAR and HT-SELEX-derived matrices to be much more successful in recognising in vivo TFBSs than PBM derived models.

Previously, the success of TF binding prediction has been found to be similar for the majority of matrices, with degenerate matrices yielding the most accurate predictions [17]. Hence researchers currently often use TF binding models from various sources indiscriminately. The notion that TF binding models obtained through different experimental methods can be variable has just recently entered the scientific discourse in a novel publication [13]. There, the authors compared PBM- and HT-SELEX-derived models and found both models to be reliable with a slight advantage of HT-SELEX models in the prediction of *in vivo* TFBSs. Our study on a larger test set (up to several thousand binding events per TF with direct comparison of models from different sources for a subset of the tested TFs) showed JASPAR to be the most reliable source for TFBS matrices in this data set.

To check if the higher accuracy of JASPAR might be the result of an overfitting of the JASPAR models to the ENCODE data, we manually checked for an overlap between the tested matrices and the ENCODE data set. For each PSSM, we studied the accompanying publication and found that this was not the case for any of the binding models included in our study. Hence we can safely assume that the current JASPAR models are indeed the most successful ones to recognise *in vivo* TFBSs.

In this study, we decided to use exonic sequences as a negative test set. Because the 5’ regions of genes occasionally contain functional binding sites [18, 19], we decided to categorically exclude the first exon of each transcript from our analyses. Thus, the percentage of functional TFBSs in the negative test set should be sufficiently decreased in order to allow computational predictions. We preferred this approach to alternative analyses, such as the usage of artificially generated random DNA sequences without TFBSs, as they might be very different from naturally occurring sequences and are also likely to increase error rates [20]. Furthermore, the usage of random exonic sequences as a negative set closely mimics the real-life research situation, therefore allowing an important insight into the limitations of current *in silico* TFBS analysis methods.

We found that the usage of only high-confidence ENCODE data leads to an increased reliability in the computerised recognition of TFBSs. However, this filtering step also introduces a potential drawback: Strong TF binding does not necessarily implicate strong TF function, as has been shown for *D. melanogaster* [21], where low-affinity binding sites can be of great importance for normal gene activation. Moreover, a researcher looking for a functional binding site *in silico* does not necessarily have any previous knowledge on the binding strength. Therefore, the decision to admit only high-confidence data into analyses – which is common practice in TF binding research [13, 17, 22]—in fact excludes a large amount of relevant information and should therefore be taken with care.

### Conservation analyses

**Fig. 3:**
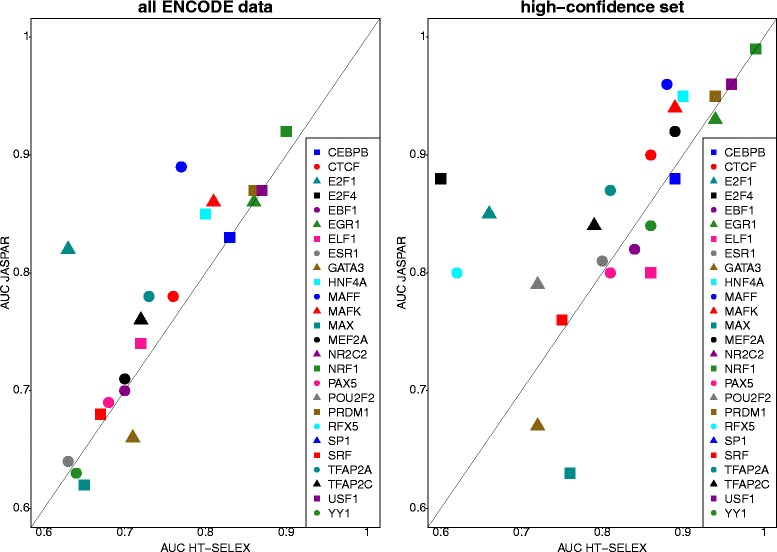
Representative plots for conservation analyses. We determined the maximum phastCons (**a**) and phyloP (**b**) scores in each experimentally confirmed binding site of BCL11A (*left panel*) and ZBTB33 (*right panel*) and calculated the averages of the maximum scores

In a real-life research situation, TFBS predictions are usually supplemented by additional analyses such as the determination of sequence conservation. We therefore further tested whether the ENCODE TFBSs (positive set) show a higher degree of conservation than the genetic background. In detail, we measured phyloP [23] and phastCons [24] scores for each position of the ENCODE TFBSs and of negative test cases (length-matched random sequences from chromosomes 1 and 2). For each TF, we then calculated the average scores of the highest conservation as well as of the average conservation score reported for each sequence. As we did not know where exactly in the ENCODE sequence the actual TFBS was located, we chose to focus on the highest conservation scores, assuming that functional TFBSs would show a high degree of conservation. However, we found that, while there was a higher degree of conservation for the ENCODE sequences, the variability in the tested sequences was very high. Moreover, we did not find a link between strong TF binding and sequence conservation because the mean maximum phyloP and phastCons scores for the high-confidence test set were not significantly higher than for all ENCODE sequences. We hence conclude that, while this may sometimes be the case, a functional binding site does not necessarily have to be highly conserved. As a representative example, Fig. 3 shows example images of conservation readings for ZBTB33 and BCL11A.

Our findings indicate that while there seems to be a certain degree of increased conservation for ChIP-seq verified TFBSs in comparison to random genetic sequences (Fig. 3), the variability between individual sites is too high to draw any reliable conclusions. These results support previous findings, which suggest that functional TFBSs can, but do not have to be evolutionarily conserved with regard to their primary nucleotide sequence [25–27]. More precisely, some researchers argue that TFBSs may fluctuate quite rapidly between species. For instance, it has been found in a comparative genomics study that the likelihood of a TFBS to be conserved between *S. cerevisiae* and its two closest relatives (*S. paradoxus* and *S. mikatae)* lies below 5 % for a majority of the sites [26]. In addition, rapid evolution of combinatorial transcription networks was found for the transcriptional regulator MCM1 in closely related fungi species [28]. Moreover, a simulation of the evolution of TFBSs by introducing local point mutations showed TFBSs and combinations of binding sites to evolve quickly [29]. Considering the fact that TFBSs are known to be relatively variable, even within the same species, it would be surprising if all functional binding sites were conserved across larger evolutionary distances [27, 30, 31]. It has also been shown in comparative studies between various species that the functionality of TFBSs can still be preserved despite underlying sequence changes [32, 33]. The three-dimensional structure of the DNA at a TFBS, including the histones, is likely to play an important role for TF binding, a notion which is slowly being acknowledged for the analysis of TFBSs [34]. As such three-dimensional structures can be well conserved while the underlying linear sequence may evolve rapidly, sequence conservation scores might not be of major importance for the assessment of TFBSs. In our study, we found that the degree of variation between individual TFBSs was too high to infer TFBSs conservation in general. Hence, measurements such as genomic location or three-dimensional conservation might be better suited for the assessment of the validity of a TFBS.

### Regulatory SNPs (rSNPs)

We compiled a set of 52 rSNPs known to directly impact TF binding or gene expression (included as Additional file 1). rSNPs are single-nucleotide polymorphisms occurring in regulatory regions such as promoters or enhancers. They are likely to be of functional significance as they do affect transcription initiation, elongation, and translational characteristics of mRNA but are more difficult to study than alterations in coding regions and are hence under-represented in the literature [35–37]. We only included alterations in this test set which have been shown to directly interfere with a TFBS’s function in in vitro or in vivo experiments. In this test set, we found differences between wild-type and mutant variants with respect to the evaluated maximum binding scores in 76 % of all observations, but they rarely exceeded 20 %. For a subset of the rSNPs, which linked to 15 different TFs (represented by 58 PSSMs from JASPAR, hPDI, and HT-SELEX), we were able to directly compare the computational predictions to the experimental findings. If solely considering maximum binding score changes, we found agreement between computational and experimental results in less than 50 % (48.1 %) of the tested matrices. However, when analysing the data with ePOSSUM (see below), we obtained a correct prediction in 62 % of the cases. Interestingly, the different binding models from the various sources again yielded quite divergent results, thus confirming strong variability in the quality of the tested matrices (see Additional file 1).

We are aware that the rSNP test set used in this study was not large enough to provide comprehensive insight. This is due to the fact that experimental data on rSNPs in TFBSs are not readily available. Hence, we decided to make the best of the available data and to provide an initial glimpse into the reliability of the state-of-the-art methods for TFBS prediction.

### ePOSSUM

Our study demonstrates that many of the existing PSSMs offer only limited reliability, while others do indeed allow reasonable predictions. In order to make our findings readily available to the scientific community, we created software called ePOSSUM that predicts the effect of genetic variation on TF binding using the matrices included in our study. It also includes a measure of significance of the assessments to indicate how reliable a prediction by a certain PSSM would be.

ePOSSUM is a web-based application that can be freely accessed at http://mutationtaster.charite.de/ePOSSUM/. Users can either enter genetic variants in the common VCF format or wild-type sequences along with the variant sequences. In the first case, the position of the variant is known and ePOSSUM scans the ENCODE ChIP-seq data used in our study for known TFBSs around this position. The existence of TFBSs at the position under study is included in the output. In total, ePOSSUM offers predictions for 81 TFs which are based on 171 different PSSMs. To speed up analysis, users can choose which TFs to include into the search for binding sites. If they submit a genomic position, they can limit the search to those TFs that are known to bind at this position.

Unlike similar applications such as motifbreakR [38], ePOSSUM does not merely subject sequences to different PSSMs, but uses their binding scores as input for a Bayes classifier. More precisely, this classifier uses the quotient of the maximum binding scores calculated for wild-type and variant sequence to determine whether the genetic alteration leads to the (predicted) gain or loss of a TFBS. As most of these quotients do not show a normal distribution, we use the R module klaR [39] for the classifier, which considers the kernel-density, i.e. the real distribution of values, to generate predictions.

Due to the poor performance of many PSSMs in discriminating real TFBSs from exonic sequences as indicated by similar maximum binding scores for both, the Bayes probability for both outcomes (TF binding versus no TF binding) is often far from 100 %. We therefore include the negative or positive predictive value (NPV/PPV) for each matrix for the given Bayes probability as a measure of the reliability of the classifier’s prediction. This value is also used to colour-code the likelihood of increased/new or decreased/lost TF binding. Darker colours represent more likely changes in TF binding. ePOSSUM uses all available PSSMs for a TF to generate a final assessment—here, only PPVs/NPVs of 70 % or above are considered. The assessment also includes whether or not a binding site for the TF was reported by ENCODE at the respective genomic location.

Different Bayes classifiers were trained for each PSSM with the quotients of all possible pairs of positive versus negative cases for the respective TF using all available sequences from our ENCODE high-confidence set as positive and exonic sequences as negative cases. The number of pairs was deliberately limited to 1,000,000 per matrix, even if more pairs were possible.

ePOSSUM is limited to human TFs and, if genomic positions were used, to genome version GRCh37. A weakness lies in the recognition of known TFBSs—as each TFBS positive ChIP-seq snippet from ENCODE comprises several hundred bases, we do not know whether or not the “real” TFBS is truly located within the fragment screened in the analysis.

## Conclusions

The assessment of variation in regulatory sequences remains a substantial challenge for the research community. While in previous decades genetic analyses focused on protein-coding regions, due to the availability of deep sequencing the search for regulatory mutations has meanwhile become a research focus. The properties of TF binding have been investigated for a long time and therefore offer a myriad of data for computational analyses. However, the utility of TF binding models for large-scale TFBS recognition has not been extensively investigated yet.

Therefore, this study is an attempt at comparing the accuracy of TF matrices obtained by various experimental methods in predicting functional TFBSs on a large scale. Thus, it enables an analysis of the quality and relevance of current TF binding prediction data.

Problems in predicting TFBSs *in silico* include high false-positive rates [16] high variability [30, 40], and insufficient knowledge of the exact in vivo binding sites [17, 41]. Current TF binding models are usually based on mere one-dimensional sequence parameters, which poses a major limitation.

To tackle these problems, researchers have to wisely choose data sources as well as TFBS predictors. Taken together, our analysis and the newly created and free-to-use software ePOSSUM provide previously unknown quality measures for computational TFBS predictions thus enabling researchers to extract and use the most meaningful of the available data.

## Methods

### TF binding models

**Fig. 4:**
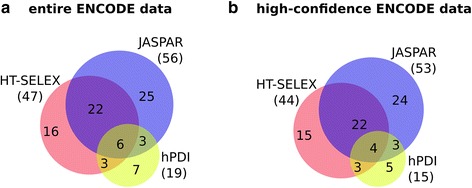
Overview of tested TFs for the entire data set (**a**) and the high-confidence data (**b**). ENCODE: Entire set of ENCODE TFBSs (2012 freeze). High confidence set: TFs reaching at least 80 % of the maximum possible binding score (published by ENCODE) in at least 100 binding instances. Please note that the intersections are not drawn to scale

TF binding matrices generated via three different experimental methodologies were obtained from the R package MotifDb [42]. This tool contains a large and up-to-date collection of freely available TF binding models. We used human PSSMs from some of the largest and most frequently used ‘open source’ TFBS data sources: hPDI (19 matrices), JASPAR (58 matrices linked with 56 different transcription factors), and HT-SELEX studies (102 matrices linked with 47 different factors). For hPDI and JASPAR, usually one matrix was available per TF, whereas the HT-SELEX data reported up to six variants per TFs. The tested PSSMs were highly variable, ranging from approximately 5 to 19 bp length, with hPDI-matrices being generally shorter than models obtained from the other two sources. In this study, in order to obtain comparable results, we only evaluated monomeric binding models, despite the fact that the latest issue of JASPAR also contains a number of homodimeric models. An overview of the binding models can be found in Fig. 4 and in the Additional file 2.

### Experimentally confirmed TFBSs (positive cases)

The positive test set consisted of ChIP-seq confirmed TF binding events, which were obtained from the ENCODE project [9] (March 2012 Freeze), as provided by the UCSC [43]. This data includes TF binding instances found in ChIP-seq experiments that had been performed on various human cell lines. For each binding event, the genomic location is reported together with a binding score indicating the strength of the protein-DNA interaction.

### High-confidence positive test set

In the first step of the experiment, all reported ChIP-seq instances were incorporated into the workflow. In a second approach, only sequences reaching at least 80 % of the maximum possible binding score published by ENCODE were included into a ‘high-confidence’ test set. Of these cases, we only tested TFs for which at least 100 binding instances were reported by ENCODE to reach this threshold. Hence, 15, 44, and 55 TFs remained for hPDI, HT-SELEX and JASPAR, respectively.

### TFBS-depleted DNA (negative test set)

For the negative test set, we used exonic sequences (GRCh37) which we obtained via Ensembl-Biomart [44]. For each sequence of the positive test set, we randomly selected an exonic sequence of equal or greater length to the corresponding TFBS sequence; in case of longer exonic sequences, these were shortened randomly to match the TFBS sequence. We routinely omitted the first exon of each transcript in order to decrease the chance of erroneously including TFBSs located downstream of the transcription start site [18, 19].

### Binding predictions

We applied each PSSM included in this study to the respective ENCODE sequences and their length-matched exonic negative controls. In total, we tested 179 models that were linked with 82 different TFs on over 4,000,000 positive and negative sequences.

Binding of a TF to a target sequence was determined using R and the Biostrings [45] function matchPWM (see Implementation). MatchPWM is a simple matching algorithm, which takes a DNA target sequence and a PWM (PSSM) of interest as inputs and computationally determines a match score. Each PWM has a theoretical maximum PWM score, which can be obtained by summing up the strongest weights. For each position of the target sequence, matchPWM compares the match score with the maximum score of the model and reports each hit above a user-defined threshold, which we set to 20 % in order to also detect ‘weak’ binding site predictions. For each of the positive and negative test set entries, we then conducted scans of both the forward and the reverse strand and determined the maximum score found within the respective pair of forward/reverse sequences. For each matched pair we thus obtained two scores, one for the true TFBS case and one for its control sequence.

The binding models in MotifDb are stored in position frequency matrices, where each column sums up to 1. MatchPWM, however, expects a position weight matrix or a position count matrix with column sums different to 1. To work around this problem, we multiplied each matrix field by the scaling factor of 100 to obtain matrices of the desired scale. As the sequences in the positive test set have been experimentally shown by the ENCODE project to contain functional TFBSs, they were expected to yield a significantly higher ratio of high match scores than their negative counterparts.

We then applied receiver operating characteristic (ROC) analyses as provided by the R package ROCR [46] to test the discriminative power of score assignments.

### Conservation analyses

Conservation scores for the three different groups of test sequences (ENCODE sequences, ENCODE high-confidence set, random length-matched control sequences) were generated using phyloP [23] and phastCons [24]. For these analyses, we obtained conservation scoring files for multiple alignments of 45 vertebrate genomes to the human genome (‘46 way’) from the UCSC PHAST package [47]. As exonic sequences are expected to be highly conserved, the negative test set for TF binding (exonic sequences, exon 1 omitted) could not be used for this analysis. Therefore, we generated a data set of random sequences by picking random genomic positions on chromosomes 1 and 2 and length-matching the sequences to ENCODE TFBS snippets.

### Regulatory SNPs (rSNPs)

As a proof of concept, we tested some of the available binding models on a small test set of 52 experimentally verified regulatory single nucleotide polymorphisms (rSNPs) that were reported in the literature. For these rSNPs the effect of the alteration on TF binding and/or gene regulation had been determined experimentally in wet-lab experiments. We collected relevant rSNP positions through an extensive literature research and then tested the corresponding binding models on a DNA sequence snippet spanning 20 bp 3’ and 5’ of the respective rSNP. Repositories or collections of verified alterations in TFBSs were found in a limited number of publications [40, 48–50], while other data was compiled manually from the literature. A summary of the rSNP test set listing the individual publications for each of the tested variants can be found in the Additional file 1.

## Implementation

### TFBS prediction

We used R version 3.1.0 and the Bioconductor suite 3.1 [51]. For the determination of TFBSs, we applied Biostrings version 2.36 [45]. In this study, we used the function matchPWM to match the PSSMs against target sequences. Human PSSMs were obtained from MotifDb version 1.10.0 [42] ROC plots were created with the R module ROCR (version 1.0-5) [46] which we also used to determine the area-under-curve (AUC) scores.

### ePOSSUM

ePOSSUM is web-based and consists of a Perl/CGI envelope for R scripts. We employ a PostgreSQL database to screen genomic positions for TFBSs reported by the ENCODE project. The R code uses the same packages as described above and the klaR module [39] which provides a kernel-density based Bayes classifier.

The connection between Perl and R is handled by the Statistics::R module.

## Additional files

Additional file 1: Test set of 52 rSNPs known to directly impact TF binding or gene expression. (XLS 13 kb) Additional file 2: Overview of tested binding models. (TXT 236 kb)

## Abbreviations

ChIP: chromatin immunoprecipitation
hPDI: human Protein-DNA Interactome
HT-SELEX: high-throughput systematic evolution of ligands by exponential enrichment
PBM: protein-binding microarray
PSSM: position-specific scoring matrix
PWM: position weight matrix
rSNP: regulatory single nucleotide polymorphism
TFBS: transcription factor binding site

## References

[1] Dynan WS, Tjian R (1983). The promoter-specific transcription factor Sp1 binds to upstream sequences in the SV40 early promoter. Cell.

[2] Lee TI, Young RA (2013). Transcriptional regulation and its misregulation in disease. Cell.

[3] Schwarz JM, Cooper DN, Schuelke M, Seelow D (2014). MutationTaster2: mutation prediction for the deep-sequencing age. Nat Methods.

[4] Sim N-L, Kumar P, Hu J, Henikoff S, Schneider G, Ng PC (2012). SIFT web server: predicting effects of amino acid substitutions on proteins. Nucleic Acids Res.

[5] Adzhubei IA, Schmidt S, Peshkin L, Ramensky VE, Gerasimova A, Bork P (2010). A method and server for predicting damaging missense mutations. Nat Methods.

[6] Stormo GD, Schneider TD, Gold L, Ehrenfeucht A (1982). Use of the “Perceptron” algorithm to distinguish translational initiation sites in E. coli. Nucleic Acids Res.

[7] Vaquerizas JM, Kummerfeld SK, Teichmann SA, Luscombe NM (2009). A census of human transcription factors: function, expression and evolution. Nat Rev Genet.

[8] Babu MM, Luscombe NM, Aravind L, Gerstein M, Teichmann SA (2004). Structure and evolution of transcriptional regulatory networks. Curr Opin Struct Biol.

[9] Gerstein MB, Kundaje A, Hariharan M, Landt SG, Yan K-K, Cheng C (2012). Architecture of the human regulatory network derived from ENCODE data. Nature.

[10] Matys V, Kel-Margoulis OV, Fricke E, Liebich I, Land S, Barre-Dirrie A (2006). TRANSFAC and its module TRANSCompel: transcriptional gene regulation in eukaryotes. Nucleic Acids Res.

[11] Xie Z, Hu S, Blackshaw S, Zhu H, Qian J (2010). hPDI: a database of experimental human protein-DNA interactions. Bioinforma Oxf Engl.

[12] Jolma A, Yan J, Whitington T, Toivonen J, Nitta KR, Rastas P (2013). DNA-binding specificities of human transcription factors. Cell.

[13] Orenstein Y, Shamir R (2014). A comparative analysis of transcription factor binding models learned from PBM. HT-SELEX and ChIP data. Nucleic Acids Res.

[14] Mathelier A, Zhao X, Zhang AW, Parcy F, Worsley-Hunt R, Arenillas DJ (2014). JASPAR 2014: an extensively expanded and updated open-access database of transcription factor binding profiles. Nucleic Acids Res.

[15] ENCODE Project Consortium (2012). An integrated encyclopedia of DNA elements in the human genome. Nature.

[16] Wasserman WW, Sandelin A (2004). Applied bioinformatics for the identification of regulatory elements. Nat Rev Genet.

[17] Weirauch MT, Cote A, Norel R, Annala M, Zhao Y, Riley TR (2013). Evaluation of methods for modeling transcription factor sequence specificity. Nat Biotechnol.

[18] Khan AH, Lin A, Smith DJ (2012). Discovery and characterization of human exonic transcriptional regulatory elements. PLoS One.

[19] Stergachis AB, Haugen E, Shafer A, Fu W, Vernot B, Reynolds A (2013). Exonic transcription factor binding directs codon choice and affects protein evolution. Science.

[20] Caballero J, Smit AFA, Hood L, Glusman G (2014). Realistic artificial DNA sequences as negative controls for computational genomics. Nucleic Acids Res.

[21] Ramos AI, Barolo S (2013). Low-affinity transcription factor binding sites shape morphogen responses and enhancer evolution. Philos Trans R Soc Lond B Biol Sci.

[22] Sikora-Wohlfeld W, Ackermann M, Christodoulou EG, Singaravelu K, Beyer A (2013). Assessing computational methods for transcription factor target gene identification based on ChIP-seq data. PLoS Comput Biol.

[23] Pollard KS, Hubisz MJ, Rosenbloom KR, Siepel A (2010). Detection of nonneutral substitution rates on mammalian phylogenies. Genome Res.

[24] Siepel A, Bejerano G, Pedersen JS, Hinrichs AS, Hou M, Rosenbloom K (2005). Evolutionarily conserved elements in vertebrate, insect, worm, and yeast genomes. Genome Res.

[25] Chen K, Rajewsky N (2007). The evolution of gene regulation by transcription factors and microRNAs. Nat Rev Genet.

[26] Doniger SW, Huh J, Fay JC (2005). Identification of functional transcription factor binding sites using closely related Saccharomyces species. Genome Res.

[27] Kasowski M, Grubert F, Heffelfinger C, Hariharan M, Asabere A, Waszak SM (2010). Variation in transcription factor binding among humans. Science.

[28] Tuch BB, Galgoczy DJ, Hernday AD, Li H, Johnson AD (2008). The evolution of combinatorial gene regulation in fungi. PLoS Biol.

[29] Stone JR, Wray GA (2001). Rapid evolution of cis-regulatory sequences via local point mutations. Mol Biol Evol.

[30] Spivakov M, Akhtar J, Kheradpour P, Beal K, Girardot C, Koscielny G (2012). Analysis of variation at transcription factor binding sites in Drosophila and humans. Genome Biol.

[31] Djebali S, Davis CA, Merkel A, Dobin A, Lassmann T, Mortazavi A (2012). Landscape of transcription in human cells. Nature.

[32] Schmidt D, Wilson MD, Ballester B, Schwalie PC, Brown GD, Marshall A (2010). Five-vertebrate ChIP-seq reveals the evolutionary dynamics of transcription factor binding. Science.

[33] Stefflova K, Thybert D, Wilson MD, Streeter I, Aleksic J, Karagianni P (2013). Cooperativity and rapid evolution of cobound transcription factors in closely related mammals. Cell.

[34] Yang L, Zhou T, Dror I, Mathelier A, Wasserman WW, Gordân R (2014). TFBSshape: a motif database for DNA shape features of transcription factor binding sites. Nucleic Acids Res.

[35] Bryzgalov LO, Antontseva EV, Matveeva MY, Shilov AG, Kashina EV, Mordvinov VA (2013). Detection of regulatory SNPs in human genome using ChIP-seq ENCODE data. PLoS One.

[36] Ponomarenko JV, Merkulova TI, Orlova GV, Fokin ON, Gorshkova EV, Frolov AS (2003). rSNP_Guide, a database system for analysis of transcription factor binding to DNA with variations: application to genome annotation. Nucleic Acids Res.

[37] Wray GA (2007). The evolutionary significance of cis-regulatory mutations. Nat Rev Genet.

[38] Coetzee SG, Coetzee GA, Hazelett DJ (2015). motifbreakR: an R/Bioconductor package for predicting variant effects at transcription factor binding sites. Bioinforma Oxf Engl.

[39] Weihs C, Ligges U, Luebke K, Raabe N, Baier D, Decker R, Schmidt-Thieme L (2005). klaR analyzing German business cycles. Data anal decis support.

[40] Laurila K, Lähdesmäki H (2009). Systematic analysis of disease-related regulatory mutation classes reveals distinct effects on transcription factor binding. In Silico Biol.

[41] Whitfield TW, Wang J, Collins PJ, Partridge EC, Aldred SF, Trinklein ND (2012). Functional analysis of transcription factor binding sites in human promoters. Genome Biol.

[42] Shannon P (2014). MotifDb: an annotated collection of protein-DNA binding sequence motifs.

[43] Raney BJ, Dreszer TR, Barber GP, Clawson H, Fujita PA, Wang T (2014). Track data hubs enable visualization of user-defined genome-wide annotations on the UCSC Genome Browser. Bioinforma Oxf Engl.

[44] Kasprzyk A (2011). BioMart: driving a paradigm change in biological data management. Database J Biol Databases Curation.

[45] Pages H, Aboyoun P, Gentleman R, DebRoy S. Biostrings: String objects representing biological sequences, and matching algorithms, 2015.

[46] Sing T, Sander O, Beerenwinkel N, Lengauer T (2005). ROCR: visualizing classifier performance in R. Bioinforma Oxf Engl.

[47] Hubisz MJ, Pollard KS, Siepel A (2011). PHAST and RPHAST: phylogenetic analysis with space/time models. Brief Bioinform.

[48] Andersen MC, Engström PG, Lithwick S, Arenillas D, Eriksson P, Lenhard B (2008). In silico detection of sequence variations modifying transcriptional regulation. PLoS Comput Biol.

[49] Buroker NE. ATF3 rSNPs, transcriptional factor binding sites and human etiology. Open J. Genet. 201;03:253.

[50] Buroker NE (2014). VEGFA rSNPs, transcriptional factor binding sites and human disease. J Physiol Sci JPS.

[51] Huber W, Carey VJ, Gentleman R, Anders S, Carlson M, Carvalho BS (2015). Orchestrating high-throughput genomic analysis with Bioconductor. Nat Methods.

