# Erratum to: A systematic, large-scale comparison of transcription factor binding site models

**DOI:** 10.1186/s12864-016-2818-8

**Published:** 2016-07-20

**Authors:** Daniela Hombach, Jana Marie Schwarz, Peter N. Robinson, Markus Schuelke, Dominik Seelow

**Affiliations:** Department of Neuropaediatrics, Charité—Universitätsmedizin Berlin, Berlin, Germany; NeuroCure Clinical Research Center, Charité – Universitätsmedizin Berlin, Berlin, Germany; Institute for Medical Genetics and Human Genetics, Charité—Universitätsmedizin Berlin, Berlin, Germany; Berliner Institut für Gesundheitsforschung/Berlin Institute of Health, Berlin, Germany

## Erratum

Unfortunately, the original version of this article [1] contained an error. Figures 2 and 3 were interchanged. Figures 2 and 3 have been corrected in the original article and are also included correctly below.

**Fig. 2:**
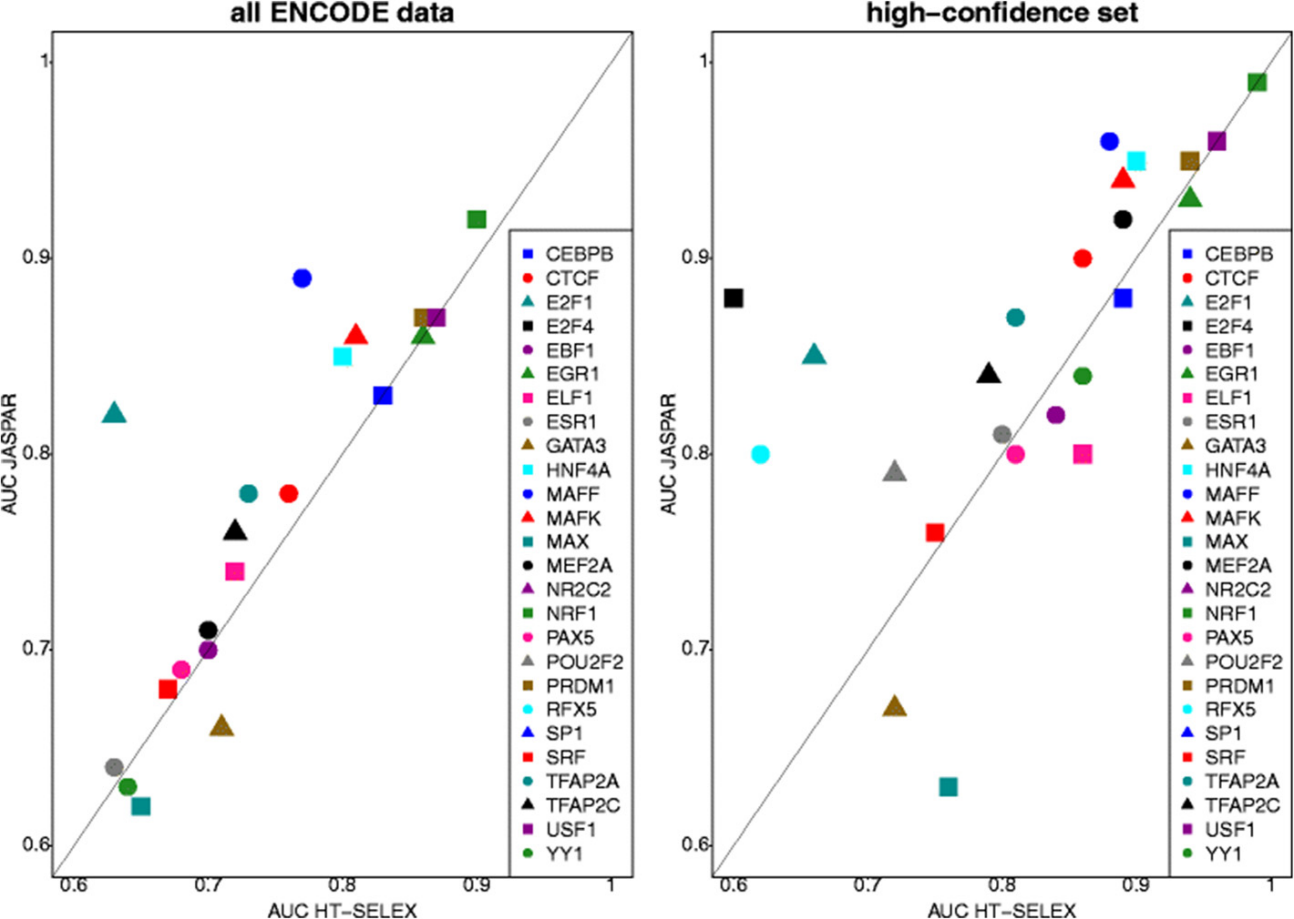
Direct comparison of binding models generated by different methods. Depicted are AUC scores for TFs stored in both JASPAR (manually collected curated models) and HT-SELEX. AUC scores were generated using ROCR. If multiple binding models were available for one TF, we depict the average AUC value

**Fig. 3:**
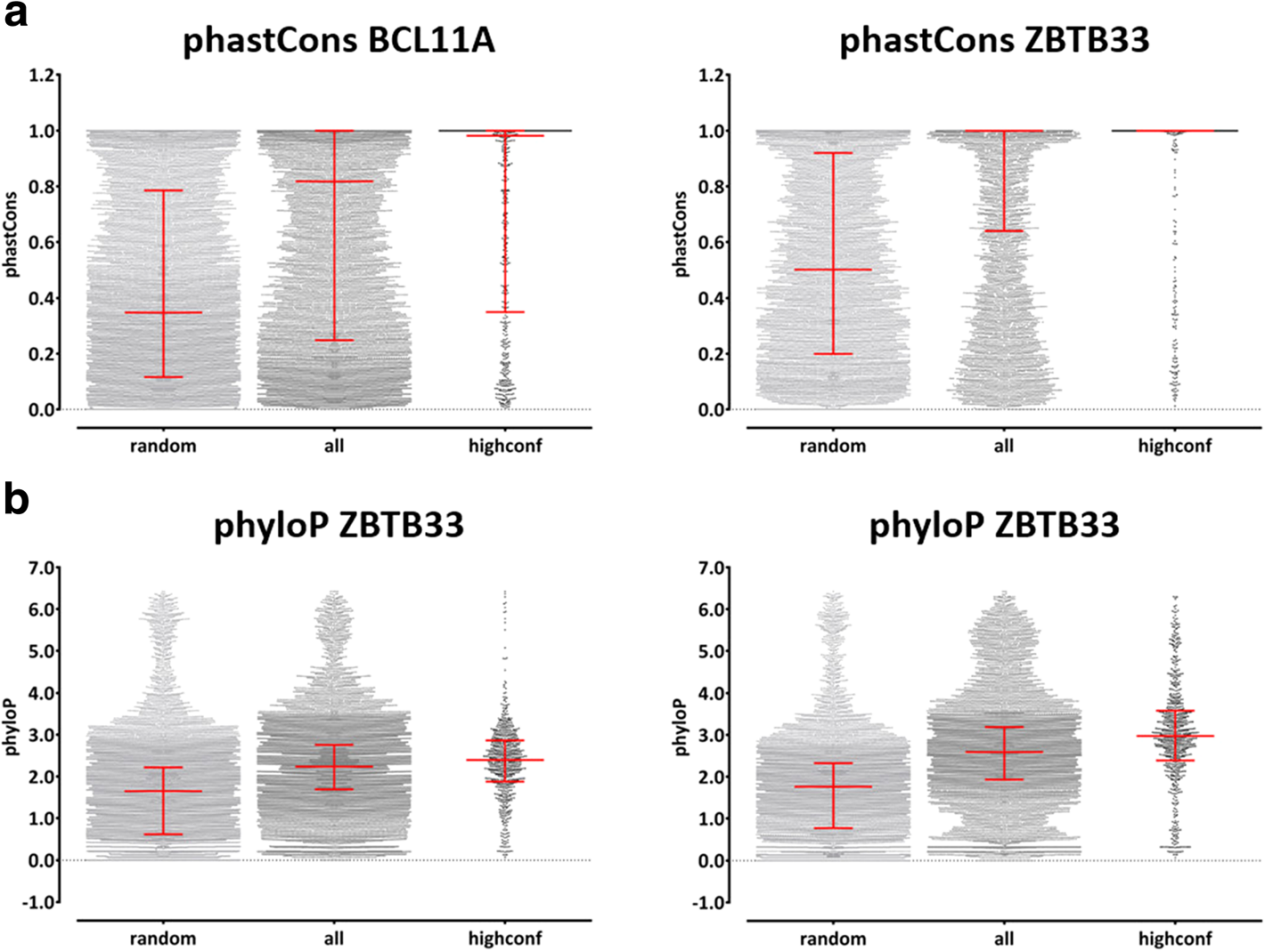
Representative plots for conservation analyses. We determined the maximum phastCons (a) and phyloP (b) scores in each experimentally confirmed binding site of BCL11A (left panel) and ZBTB33 (right panel) and calculated the averages of the maximum scores
